# Qualitative Evaluation of Common Quantitative Metrics for Clinical Acceptance of Automatic Segmentation: a Case Study on Heart Contouring from CT Images by Deep Learning Algorithms

**DOI:** 10.1007/s10278-021-00573-9

**Published:** 2022-01-26

**Authors:** L. B. van den Oever, W. A. van Veldhuizen, L. J. Cornelissen, D. S. Spoor, T. P. Willems, G. Kramer, T. Stigter, M. Rook, A. P. G. Crijns, M. Oudkerk, R. N. J. Veldhuis, G. H. de Bock, P. M. A. van Ooijen

**Affiliations:** 1 Department of Radiation Oncology, University Medical Center Groningen, University of Groningen, Hanzeplein 1, 9713GZ Groningen, The Netherlands; 2 Department of Surgery, University Medical Center Groningen, University of Groningen, Hanzeplein 1, 9713GZ Groningen, The Netherlands; 3 Department of Radiology, University Medical Center Groningen, University of Groningen, Hanzeplein 1, 9713GZ Groningen, The Netherlands; 4 Department of Radiology, Martini Hospital, Van Swietenplein 1, 9728 NT Groningen, The Netherlands; 5 Faculty of Medical Sciences, University of Groningen, Groningen, The Netherlands; 6 Department of Electrical Engineering, Computer Science and Mathematics, University of Twente, Drienerlolaan 5, 7522 NB Enschede, The Netherlands; 7 Department of Epidemiology, University Medical Center Groningen, University of Groningen, Hanzeplein 1, 9713GZ Groningen, The Netherlands

**Keywords:** Automatic contouring, Deep learning, CT, Qualitative assessment, Turing test

## Abstract

Organs-at-risk contouring is time consuming and labour intensive. Automation by deep learning algorithms would decrease the workload of radiotherapists and technicians considerably. However, the variety of metrics used for the evaluation of deep learning algorithms make the results of many papers difficult to interpret and compare. In this paper, a qualitative evaluation is done on five established metrics to assess whether their values correlate with clinical usability. A total of 377 CT volumes with heart delineations were randomly selected for training and evaluation. A deep learning algorithm was used to predict the contours of the heart. A total of 101 CT slices from the validation set with the predicted contours were shown to three experienced radiologists. They examined each slice independently whether they would accept or adjust the prediction and if there were (small) mistakes. For each slice, the scores of this qualitative evaluation were then compared with the Sørensen-Dice coefficient (DC), the Hausdorff distance (HD), pixel-wise accuracy, sensitivity and precision. The statistical analysis of the qualitative evaluation and metrics showed a significant correlation. Of the slices with a DC over 0.96 (*N* = 20) or a 95% HD under 5 voxels (*N* = 25), no slices were rejected by the readers. Contours with lower DC or higher HD were seen in both rejected and accepted contours. Qualitative evaluation shows that it is difficult to use common quantification metrics as indicator for use in clinic. We might need to change the reporting of quantitative metrics to better reflect clinical acceptance.

## Introduction

The number of papers published on deep learning (DL) in the medical imaging field is rising rapidly each year [1]. However, it can be difficult to implement the results of this type of research in clinic, due to difficulty in interpreting validation studies. For segmentation tasks, various overlap metrics are used to describe the accuracy of neural networks, and, commonly, a range of random overlap metrics is reported. Recently, guidelines on the reporting of accuracy metrics have been published to reduce this variance of reported metrics in artificial intelligence (AI) challenges [2]. These overlap metrics are calculated by comparing the ‘ground truth’ with the prediction of the AI software. Although high values of these might be achieved, it could still mean that the predictions of a neural network might be unsuited for clinical use [3]. For this purpose, this paper will investigate the correlation of the three most important metrics and the acceptance for clinical use of a deep learning algorithm by doing a qualitative assessment. The focus of this paper is the qualitative assessment; therefore, we selected a use case, automatic heart contouring and a random AI method.

Automatic heart contouring for radiotherapeutic planning will be used as an example of an AI algorithm. Radiotherapeutic planning is labour intensive due to its need for manual segmentations of various organs surrounding the organ targeted. Therefore, automatic methods for segmenting organs on CT images have been an attractive field of research. More recent developments in this field use deep learning algorithms to attempt to automate this process and to reduce the workload of radiotherapists [4–6]. Automatic heart contouring can also be used for improving the accuracy of heart disease detection AI algorithms by cropping the images around the heart, thereby removing irrelevant parts of the image that could otherwise lead to bias in an algorithm. For instance, in the detection of coronary artery calcium (CAC), false positive plaques are sometimes found outside of the heart. By using a heart contour, these false positives could be further reduced [7].

A way of investigating the readiness of an AI algorithm is to validate its accuracy in a clinical setting. In the case of segmentation results, the contours could be shown to trained readers to do a qualitative evaluation [8]. When the segmentation results are assessed by clinicians for correctness, the relation to the geometrical evaluation metrics can be explored to see if these can somehow be linked to clinical usability. This paper will test if the five most important metrics to measure overlap could be used to indicate if AI algorithms are fit for clinical practice.

## Materials and Methods

### Nomenclature

The terminology coined by Liu et al. [9] is used for the description of our datasets. The training dataset is the dataset used for optimization of the model weights, the tuning dataset is used to avoid overfitting during the training phase, and the internal validation dataset is used for measuring the accuracy of the neural networks after training.

### Population

The dataset used in this paper comes from the Thoraces retrospective study composed of nearly 4000 female breast cancer patients treated between 2005 and 2008 at the Department of Radiation Oncology of the University Medical Center Groningen. Exclusion criteria included the unavailability of a planning CT scan or a medical history of cancer requiring adjuvant treatment [10]. A total of 377 CT volumes with heart delineations available were randomly selected for our study. These 377 volumes were divided into a training dataset (303 volumes), a tuning dataset (37 volumes) and an internal validation dataset (37 volumes).

### Scan Protocol

Non-triggered CT scans were acquired from a SOMATOM Sensation Open (40 slice, Siemens Medical Inc.). The tube voltage and current were 140 kVp and 100 reference mAs, respectively. The slice thickness was either 5 or 3 mm, depending on the year the scans were made.

### Data Annotation and Processing

The CT scans of the patients were automatically contoured by using a heart atlas registration software (Mirada RTx 1.6 and Workflow Box 1.4, Mirada Medical Ltd., Oxford, United Kingdom, research only). The atlas was based on 20 breast cancer patients [10, 11]. The contouring of these patients was checked and, if needed, corrected by a senior radiotherapy technician and subsequently validated by a radiation oncologist.

For use in deep learning, the CT images and the labels were deidentified, extracted and converted to NumPy format files. The CT images were windowed to mediastinum window level (W: 350 HU, L: 50 HU). The slices were reconstructed into volumes to extract sagittal and coronal slices for use in the 2.5D network.

The dataset was inherently skewed due to the heart not being included in most of the CT slices. To balance the dataset, all the CT slices containing the heart were included, while only 20% of slices without heart were included. This results in 12,620 axial CT slices, 38,973 coronal CT slices and 49,147 sagittal CT slices in total for training.

### Architectures and Model Training

The architecture of the neural network used in this paper is based on U-net [12]. We have added dilated convolutional layers in the first two and last two convolutional layers of the original network. This allows the network to make more use of spatial information in the larger images. One neural network is trained for axial, sagittal and coronal slices each, for a total of 3 neural networks. The final prediction is created by combining the predicted contours of the three neural networks with either a majority voting operator.

The last activation layer was used a sigmoid function. All neural networks are trained for 20 epochs with a batch size of 5 images. The optimizer used was Adam with a learning rate of 10^−5^. The 1-Dice Similarity Coefficient (DC) is used as the loss function for the 2.5D U-nets. Training was performed on the Peregrine cluster of the University of Groningen using an NVIDIA V100 Tensor Core GPU. The code was implemented in Python v3.6 and used TensorFlow v2 and Keras v2.6.

### Metrics

To assess the results of the experiment performed in this paper, the DC, precision, sensitivity, pixel-wise accuracy and 95% Hausdorff distance (95% HD) of the predictions of the internal validation dataset were calculated. The predictions are created by a majority voting operator between the three predictions of the axial, sagittal and coronal networks. The DC is a measure of the amount of overlap between the prediction mask and the ground truth mask. The 95% HD is based on the maximum Hausdorff distance. This metric calculates the maximum distance of a predicted contour point to the nearest contour point of the ground truth. By using the 95th percentile, outliers have less impact. Therefore, higher values mean more distance between the edges of the ground truth and the predictions, and as such, worse results. The precision and sensitivity give insight on the false positive voxels, true positive voxels and true negative voxels predicted by the networks. Finally, pixel-wise accuracy was calculated for overall accuracy.

This paper uses median values in reporting the metrics. It is common to see mean values reported in papers, but the accuracy metrics are not normally distributed and should, therefore, be reported as medians.

### Qualitative Evaluation

**Fig. 1:**
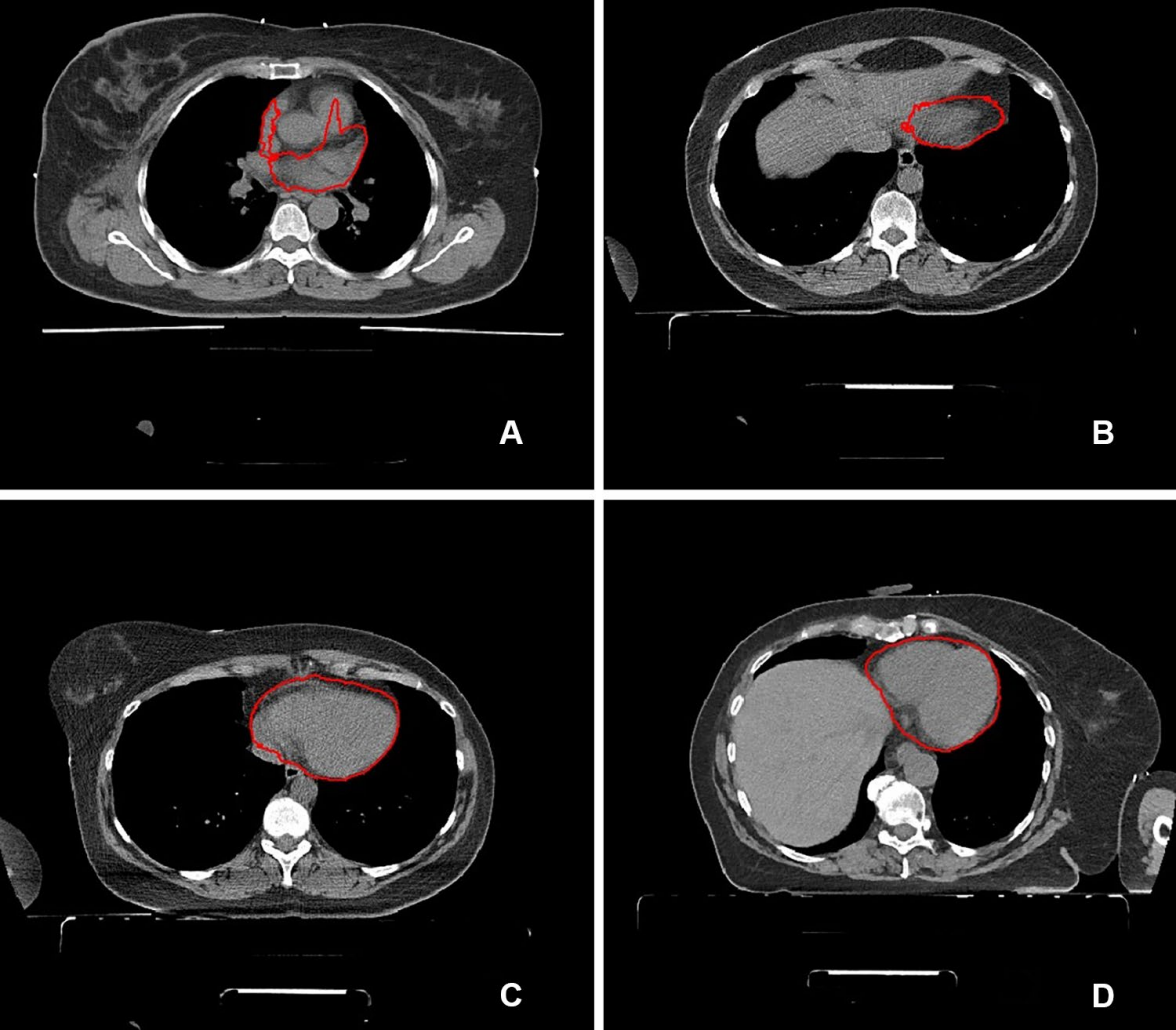
4 CT slices with predicted contouring of the heart as shown to the radiologists for the qualitative evaluation. The letters correspond with the consensus answers for this particular slide. **A** was rejected with clear mistakes, **B** was rejected with minor but clinically relevant mistakes, **C** was accepted with small but irrelevant mistakes and **D** was accepted with no mistakes

To evaluate the correlation between the metrics and acceptance of slices in clinical applications, three experienced radiologists, specialized in cardiothoracic imaging, scored 101 slices from the internal validation dataset with predicted contours as shown in Fig. 1. The scoring was done separately, so all slices had separate answers per radiologist. The answers available to the question ‘Your colleague asks you to check the following contour, would you:’ were:

- A, adjust the contour, there are clear major mistakes
- B, adjust the contour, there are minor but clinically relevant mistakes,
- C, accept the contour, there are small but irrelevant mistakes
- D, accept the contour, it is very accurate [11]

The final answer was made by majority vote. For further analysis, slices where the consensus was A or B were assigned as rejected slices, whereas C and D consensus were treated as accepted slices. Every slice, therefore, had a quantitative number based on the evaluation metrics and had a qualitative measure in the form of accepted or rejected. This was done to investigate the validity of a quantitative metric threshold for accepting or rejecting contours.

A threshold for the evaluation metric was then used to divide the slices in accepted and rejected slices. Cohen’s kappa could be calculated to investigate the agreement between the readers and the use of a metric for accepting or rejecting slices.

### Statistical Analysis

The differences between the values of the metrics and the evaluation answers of the readers were analysed using a Kruskal–Wallis H test in SPSS. A Kruskal–Wallis H test was selected to compensate for the small dataset and the skewed distribution of the data.

## Results

The 2.5D CNN achieved a DC of 0.91, a precision of 0.89, a sensitivity of 0.94, pixel-wise accuracy of 0.95 and 95% HD of 12.8 voxels with the majority voting as method for combining the 3 predictions.

### Qualitative Evaluation

**Fig. 2:**
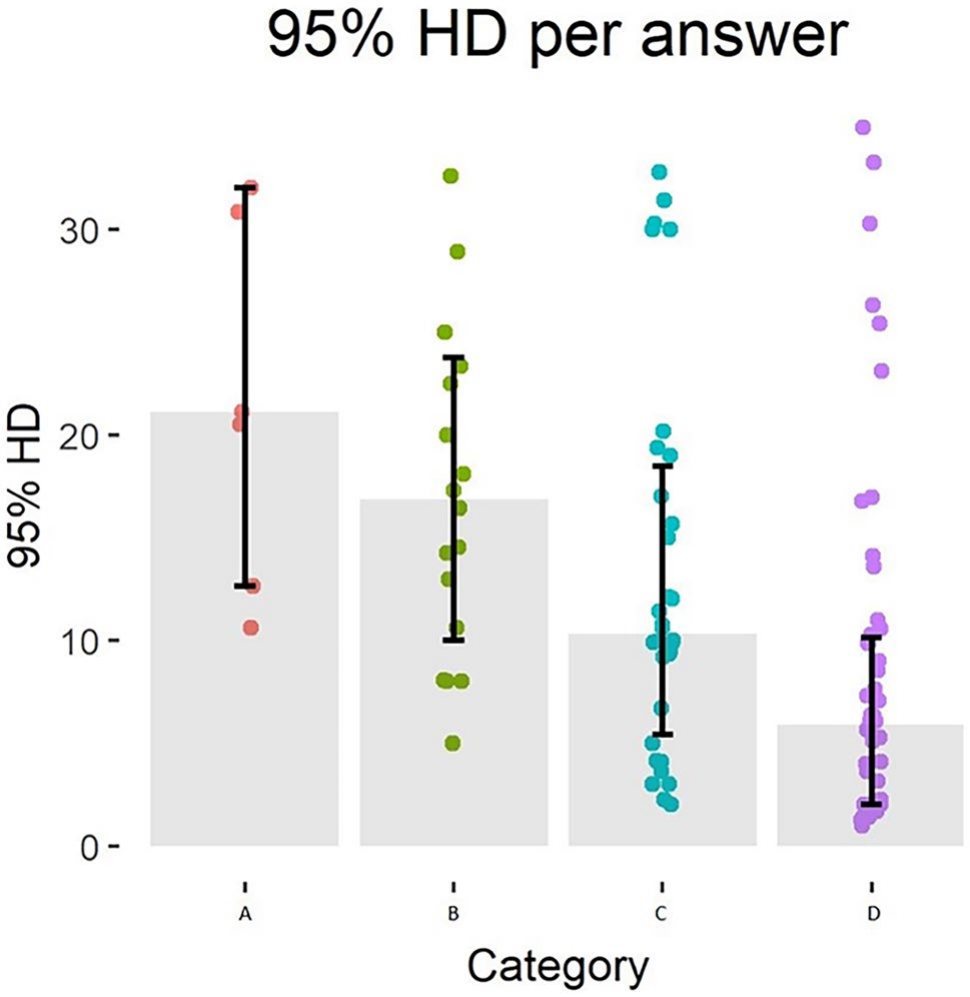
Overview of the consensus answers and the corresponding 95% HD values of the slices. The grey bar indicates the median value with the 25th and 75th quartiles. The coloured dots are the qualitative evaluation answers

**Fig. 3:**
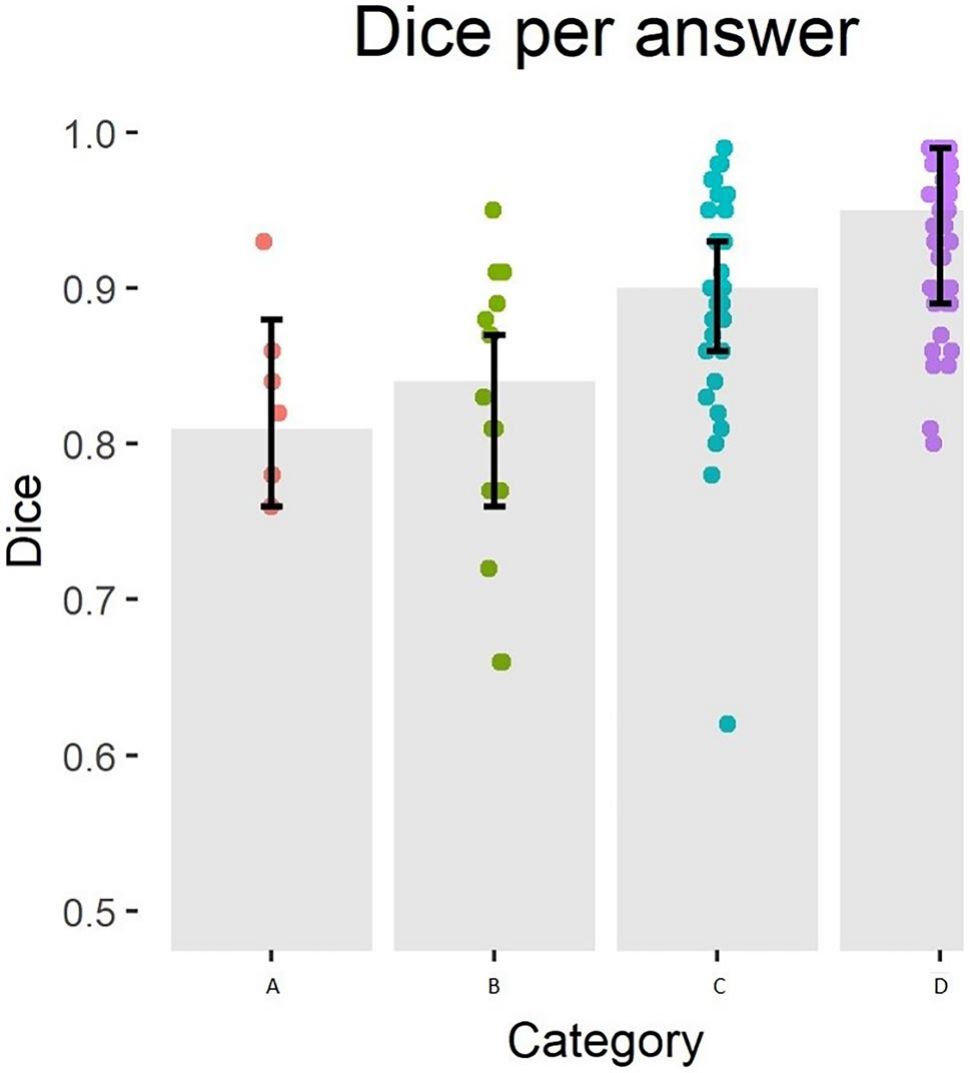
Overview of the consensus answers and the corresponding DC values of the slices. The grey bar indicates median value with the 25th and 75th quartiles. The coloured dots are the qualitative evaluation answers

**Fig. 4:**
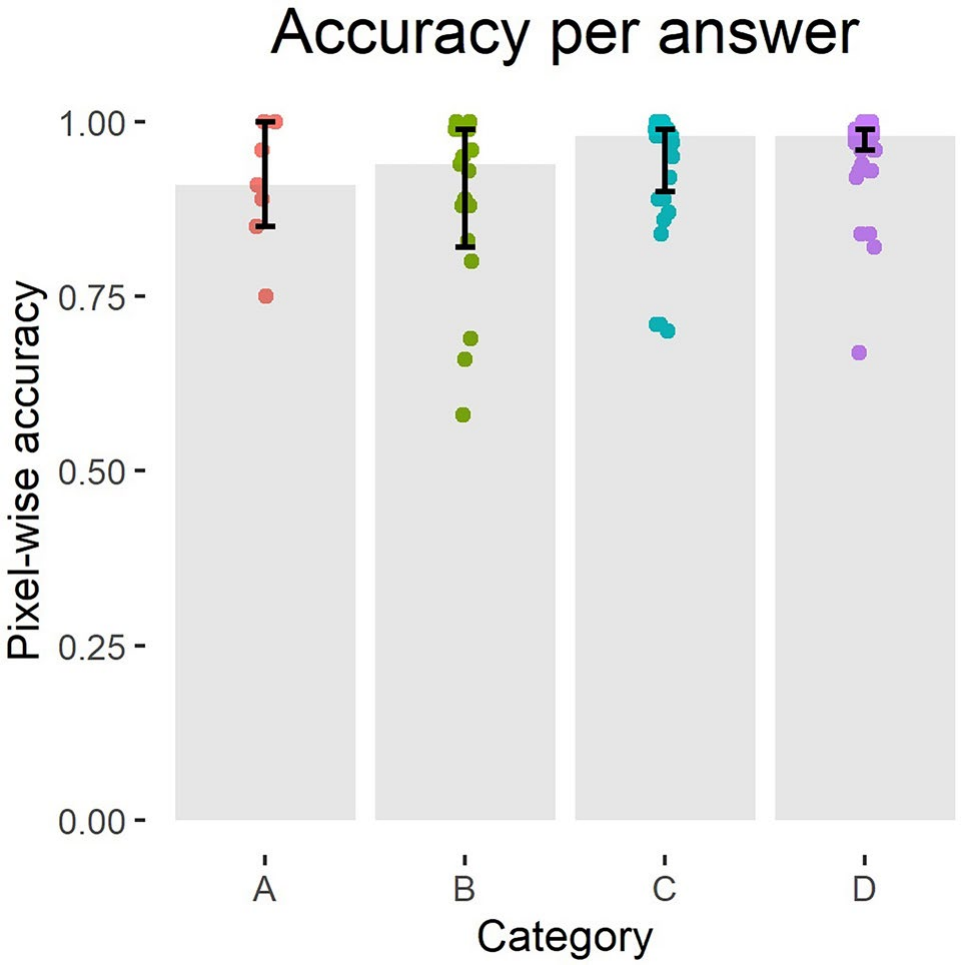
Overview of the consensus answers and the corresponding pixel-wise accuracy values of the slices. The grey bar indicates median value with the 25th and 75th quartiles. The coloured dots are the qualitative evaluation answers

Three radiologists scored 101 CT slices with predicted contours for correctness of the contour. Seventy-six slices were accepted and 25 rejected. The median values and interquartile ranges of the overlap metrics and pixel-wise precision, sensitivity and accuracy grouped either per rejected/accepted or per answer can be seen in Tables 1 and 2. Figures 2, 3 and 4 show the consensus answers and their correlating 95% HD, DC and pixel-wise accuracy values of the CT slices. Of the slices with a DC over 0.96 (*N* = 20) or a 95% HD under 5 voxels (*N* = 25), no slices were rejected by the readers. The pixel-wise accuracy showed no such threshold.

**Table 1:** Results of the evaluation as done by the radiologists. The answers are combined by majority vote and grouped by rejected (A or B answers) or accepted (C or D answers) slices. The medians and 25th and 75th quartiles of the DC, precision, sensitivity, 95% HD and pixel-wise accuracy are given for the accepted and rejected slices

	DC	95% HD (in voxels)	Precision	Sensitivity	Pixel-wise accuracy
**Rejected (*N* = 25)**	0.83 (0.76–0.87)	18.1 (11.7–27.0)	0.80 (0.72–0.85)	0.87 (0.80–0.97)	0.93 (0.84–0.99)
**Accepted (*N* = 76)**	0.93 (0.87–0.97)	8.08 (3.27–13.2)	0.90 (0.82–0.98)	0.98 (0.95–0.99)	0.98 (0.95–0.99)

**Table 2:** Results of the evaluation by radiologists per answer. The medians and 25th and 75th quartiles of the metrics are given per answer

**Consensus**	DC	95% HD (in voxels)	Precision	Sensitivity	Pixel-wise accuracy
**A (*N* = 7)**	0.81 (0.76–0.88)	21.1 (12.7–32.0)	0.72 (0.71–0.91)	0.89 (0.84–0.98)	0.91 (0.85–1.00)
**B (*N* = 18)**	0.84 (0.76–0.87)	16.9 (10.0–23.8)	0.81 (0.73–0.85)	0.86 (0.78–0.96)	0.94 (0.82–0.99)
**C (*N* = 32)**	0.90 (0.86–0.93)	10.3 (5.4–18.5)	0.87 (0.77–0.94)	0.98 (0.77–0.94)	0.98 (0.90–0.99)
**D (*N* = 44)**	0.95 (0.89–0.99)	5.90 (2.00–10.2)	0.94 (0.86–0.99)	0.99 (0.97–0.99)	0.98 (0.96–0.99)

**Fig. 5:**
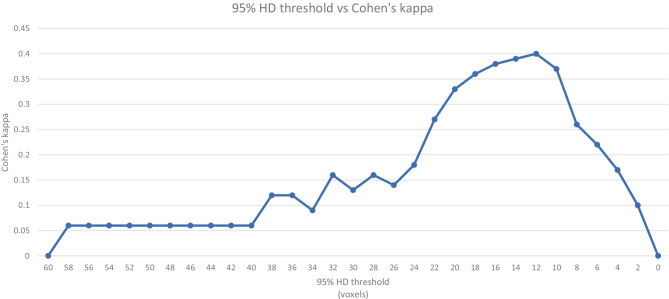
This graph shows the agreement between metrics and readers when shifting the threshold for the 95% HD for acceptance or rejection of contours

**Fig. 6:**
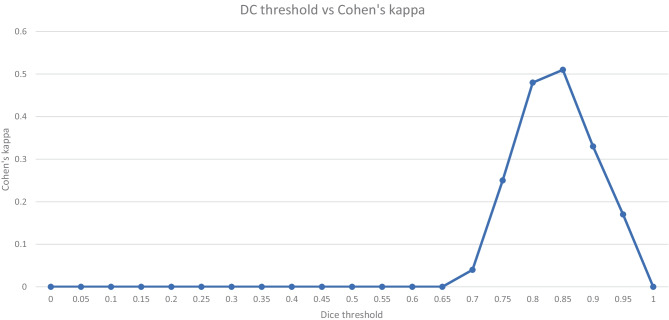
This graph shows the agreement between metrics and readers when shifting the threshold for the DC for acceptance or rejection of contours

**Fig. 7:**
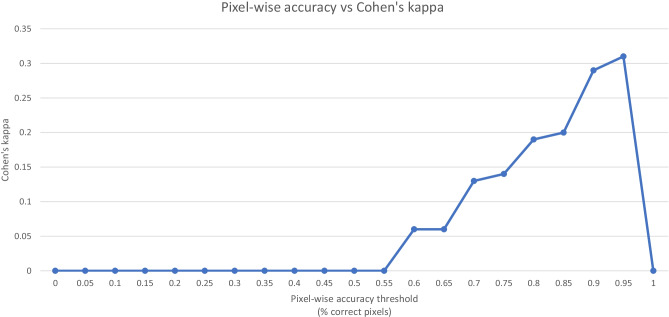
This graph shows the agreement between metrics and readers when shifting the threshold for the pixel-wise accuracy for acceptance or rejection of contours

The highest agreement between thresholding the metrics and the consensus of the readers is reached at thresholds for the 95% HD under 12, a DC of over 0.85 and a pixel-wise accuracy of over 0.96 with Cohen’s kappa of 0.40, 0.51 and 0.31 respectively (Figs. 5, 6 and 7). Precision and sensitivity reached Cohen’s kappa of 0.36 and 0.47.

### Statistical Analysis

The Kruskal–Wallis H test showed that there was a statistically significant difference in all 4 quantative metrics compared to the consensus answers with *χ*^2^s of 37.0, 23.6, 23.2 and 27.7, all *p* < 0.001, for the DC, precision, sensitivity and 95% HD metrics and critical *χ*^2^ of 7.81. Pixel-wise accuracy showed no statistically significant difference between the metrics and consensus answers with a *χ*^2^ of 4.82.

## Discussion

We show that the DC, precision, sensitivity and 95% HD metrics correlate with clinical acceptance by using a Kruskal–Wallis H test. However, the results also show that a high DC or low 95% HD is no guarantee for clinical acceptance unless in the higher echelons for the DC and lower echelons for the 95% HD. Based on our findings, it is difficult to indicate clinical acceptance based on a minimal value of a metric. An argument could be made that a minimum of 0.96 for the DC and a maximum of 5 for the HD, for every prediction, would ensure clinical acceptability. This would require a very stable and generalized deep learning algorithm.

The automatic contouring of the whole heart, albeit promising, still needs to be corrected, although often minimally, even with high metric scores. It would, however, be usable as a first-stage contouring that can then be corrected by the technicians. The average DC achieved shows that most slices would need correction. Only 4 of the 50 CT slices shown to the radiologists with a DC of over 0.9 were rejected.

### Limitations

There are a number of limitations to the work presented in this paper. No postprocessing was performed on the predictions of the neural networks. Small postprocessing steps, such as the removal of small objects or the smoothing of edges, such as seen in the right most image of Fig. 1, might have improved the accuracy further. We also recommend doing this when presenting contours to readers. A number of contours were rejected only due to the edges being rough in the contours. These might not be clinically significant and easily removed with postprocessing steps.

**Fig. 8:**
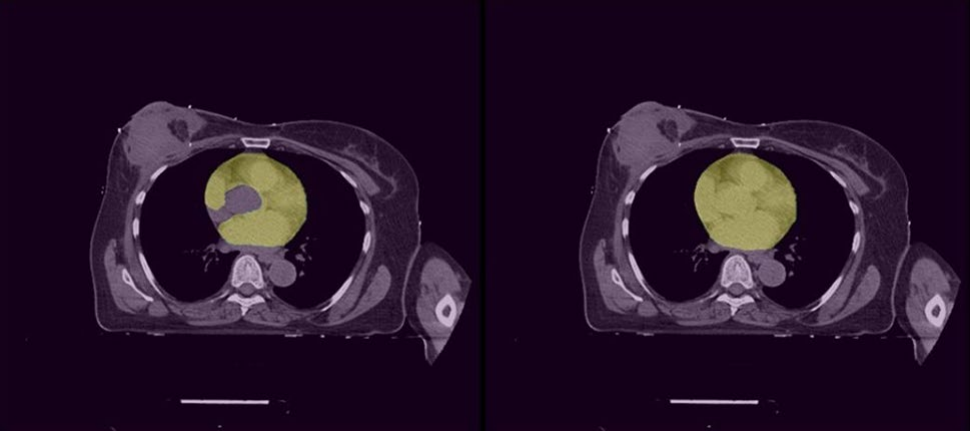
An example of mismatch between readers and the ground truth. On the left, a ground truth segmentation can be seen with excluded aorta. On the right, the predicted segmentation can be seen

The ground truth masks of the heart contours did not include the aorta. The DL algorithms, therefore, learned to exclude the aorta as well. For the qualitative evaluation, however, it was difficult for the readers to evaluate if the aorta was in the CT slice in some images. The readers were only presented with random CT slices, so could not scroll through the volume to evaluate the aorta based on surrounding slices. Ten slices were accepted with low DC and high HD due to this effect (Fig. 8).

Another limitation is that this work is only tested on a single dataset with large ground truths and with only one AI architecture. It would be interesting to investigate the effects in smaller or more difficult segmentation problems and the effect of different AI tools or architectures. It should be noted that the effect of very high DC or very low 95% HD on clinical acceptance is only based on a very small sample, only 20 and 25 slices in the cases of the DC and 95% HD metric respectively. Similarly, only three metrics were analysed for their clinical acceptability.

### Implications for Clinic and Future Work

With this work, we hope to make a step towards implementation of AI software into the clinic by showing the relevance of qualitative evaluation of clinical acceptance and the necessity of better evaluation metrics. Since average values of metrics seem to fail in predicting the use in clinic, we would like to suggest minimal or maximal values of metrics were reported. This would, of course, be susceptible to outliers, so an idea to minimize that might be to use minimal value of metrics and excluding a certain percentage of outliers. If a method could be found for indicating clinical usability of an AI algorithm, it might be easier for clinicians to interpret results of AI papers.

## Abbreviations

CAC: Coronary artery calcium
AI: Artificial intelligence
CT: Computed tomography
DC: Dice similarity coefficient
HD: Hausdorff distance
DL: Deep learning
CNN: Convolutional neural network
